# Efficient Detection of Mediterranean β-Thalassemia Mutations by Multiplex Single-Nucleotide Primer Extension

**DOI:** 10.1371/journal.pone.0048167

**Published:** 2012-10-26

**Authors:** Biljana Atanasovska, Georgi Bozhinovski, Dijana Plaseska-Karanfilska, Lyubomira Chakalova

**Affiliations:** 1 Research Center for Genetic Engineering and Biotechnology, Macedonian Academy of Sciences and Arts, Skopje, Former Yugoslav Republic of Macedonia; 2 Department of Molecular Biology of the Cell Cycle, Institute of Molecular Biology, Bulgarian Academy of Sciences, Sofia, Bulgaria; Instituto de Ciencia de Materiales de Madrid - Instituto de Biomedicina de Valencia, Spain

## Abstract

β-Thalassemias and abnormal hemoglobin variants are among the most common hereditary abnormalities in humans. Molecular characterization of the causative genetic variants is an essential part of the diagnostic process. In geographic areas with high hemoglobinopathy prevalence, such as the Mediterranean region, a limited number of genetic variants are responsible for the majority of hemoglobinopathy cases. Developing reliable, rapid and cost-effective mutation-specific molecular diagnostic assays targeting particular populations greatly facilitates routine hemoglobinopathy investigations. We developed a one-tube single-nucleotide primer extension assay for the detection of eight common Mediterranean β-thalassemia mutations: Codon 5 (-CT); CCT(Pro)->C–, Codon 6 (-A); GAG(Glu)->G-G, Codon 8 (-AA); AAG(Lys)->–G, IVS-I-1 (G->A), IVS-I-6 (T->C), IVS-I-110 (G->A), Codon 39 (C->T), and IVS-II-745 (C->G), as well as the hemoglobin S variant beta 6(A3) Glu>Val. We validated the new assay using previously genotyped samples obtaining 100% agreement between independent genotyping methods. Our approach, applicable in a range of Mediterranean countries, offers a combination of high accuracy and rapidity exploiting standard techniques and widely available equipment. It can be further adapted to particular populations by including/excluding assayed mutations. We facilitate future modifications by providing detailed information on assay design.

## Introduction

Hemoglobinopathies are the most abundant group of monogenic abnormalities in humans representing a health burden comparative to that of other major diseases [1], [2]. They are caused by genetic defects affecting the globin genes encoding for the hemoglobin α and β chains. In particular, a great variety of mutations disturb the function of the *β*-globin *HBB* gene [3], [4]. The majority of the genetic variations are located within or in close proximity to the gene and can be present in heterozygous, homozygous and compound heterozygous states. Many *HBB* mutations have been shown to reduce or abolish the expression of the *HBB* gene from the abnormal chromosome leading to net hemoglobin deficiency and β-thalassemia. Thalassemia patients suffer from anemia and pathological complications, such as splenomegaly, skeletal abnormalities and growth retardation. Depending on the necessity for transfusions in managing the disease, β-thalassemia is clinically classified as minor, intermedia or major. Untreated β-thalassemia major often leads to death in early childhood. In contrast, heterozygotes for mutations associated with mild phenotypes can lead a normal life and remain unaware of their carrier status facing a high chance of having children with β-thalassemia if their partners are also carriers of the trait. Some geographic areas including the Mediterranean region have high prevalence of β-thalassemias [5]. Another subclass of *HBB* mutations, rather than decreasing the amount of β chain as in β-thalassemia, lead to the production of abnormal hemoglobin variants. One of the most widespread abnormal hemoglobins worldwide is hemoglobin S (HbS). The defect results from a single amino acid substitution in the β-globin chain leading to an increased propensity for polymerization of hemoglobin into long chains that distort red blood cells. Homozygous patients suffer from sickle cell disease, a serious condition with numerous complications including acute hemolytic and vaso-occlusive crises [1].

Laboratory identification of the underlying genetic defects provides definitive hemoglobinopathy diagnosis and allows for genetic counseling of affected families including the option for prenatal diagnostics. Routine molecular investigations have consequently become an integral part of the diagnostic protocols in many laboratories. It is essential to develop rapid, inexpensive and reliable molecular diagnostic procedures. However, the heterogeneous nature of the diseases presents a substantial challenge. Only a handful of methods are suitable for unbiased detection of large groups of mutations including previously undescribed genetic variants. These include chemical cleavage of mismatch analysis [6], mismatch-specific endonuclease analysis [7], single-strand conformation polymorphism electrophoresis [8], denaturing gradient gel electrophoresis [9], temporal temperature gel electrophoresis [10]. In recent years, direct DNA sequencing of the *HBB* gene has become more common [11], [12]. These assays are essential for the identification of rare and novel mutations. However, a limited number of mutations accounts for the majority of hemoglobinopathies in any given population. Thus, for routine screening of well characterized populations, mutation-specific strategies are often preferred [11]. Commonly used techniques include restriction endonuclease analysis [13], multiplex amplification refractory mutation system polymerase chain reaction (PCR) [14], [15], allele-specific oligonucleotide (ASO) hybridization [16]–[20], reverse dot-blot [18], [21], [22], allele-specific PCR [23], high-resolution melting [24], array-based technologies [22], [25]–[30], primer extension assays [12], [31]–[35]. The latter three technologies offer the highest potential for automation. In particular, multiplex fluorescence-based primer extension, also referred to as minisequencing, is dependable and suitable for scaling up for high-throughput applications [31], [32].

Until recently, the primary method for identification of β-thalassemia mutations in our laboratory was ASO hybridization with mutation-specific probes [17], [36]. We were looking to reduce the average time necessary for reaching a diagnosis by switching to a highly reliable, semi-automated technique allowing simultaneous detection of the most commonly occurring mutations. A review of the published methods for detection of pre-defined sets of Mediterranean mutations revealed the need to develop a new strategy. Here we report a multiplex assay specific for common Mediterranean *HBB* genetic variants including 3 microdeletions and 6 point mutations: Codon 5 (-CT), Codon 6 (-A), beta 6(A3) Glu>Val, Codon 8 (-AA), IVS-I-1 (G->A), IVS-I-6 (T->C), IVS-I-110 (G->A), Codon 39 (C->T), and IVS-II-745 (C->G). Our protocol utilizes PCR amplification of a single *HBB* fragment spanning all of the examined mutations followed by multiplex single-nucleotide primer extension with fluorescently labeled dideoxynucleotides. Our primer extension set includes oligonucleotides hybridizing next to the variant nucleotides on both genomic strands ensuring double interrogation of the bases of interest in a single reaction. Extension products are analyzed by automated capillary electrophoresis. We present a cost-effective molecular diagnostic tool that can be applied in a number of Mediterranean countries.

## Results

### Multiplex Single-nucleotide Primer Extension Assay: Optimization and Validation

The selection of target mutations is an important consideration affecting the applicability of the method. Our choices were based purely on mutation prevalence in our target population comprising patients from Macedonia and several neighboring countries [37]–[39]. We took advantage of the extensive genetic information collected through hemoglobinopathy diagnostics in our laboratory in order to design a mutation-specific assay custom-tailored for our purposes. We selected the top eight most common β-thalassemia mutations to include in the minisequencing assay (Table 1 and Figure S1). The deleted nucleotide in Codon 6 (-A) coincides with the variable nucleotide in the beta 6(A3) Glu>Val hemoglobin variant so the HbS mutation also became part of the mutation panel.

**Table 1:** Panel of assayed *HBB* genetic variants.

Mutation name^a^	HGVS nomenclature^b^	Type of hemoglobinopahy
Codon 5 (-CT); CCT(Pro)->C-	HBB:c.17_18delCT	beta0 thalassemia
Codon 6 (-A); GAG(Glu)->G-G	HBB:c.20delA	beta0 thalassemia
beta 6(A3) Glu>Val	HBB:c.20A>T	sickle cell disease
Codon 8 (-AA); AAG(Lys)->-G	HBB:c.25_26delAA	beta0 thalassemia
IVS-I-1 (G->A)	HBB:c.92+1G>A	beta0 thalassemia
IVS-I-6 (T->C)	HBB:c.92+6T>C	beta+ thalassemia
IVS-I-110 (G->A)	HBB:c.93−21G>A	beta+ thalassemia
Codon 39 (C->T)	HBB:c.118C>T	beta0 thalassemia
IVS-II-745 (C->G)	HBB:c.316−106C>G	beta+ thalassemia

a Huisman et al. [3].

b Patrinos et al. [4].

**Figure 1:**
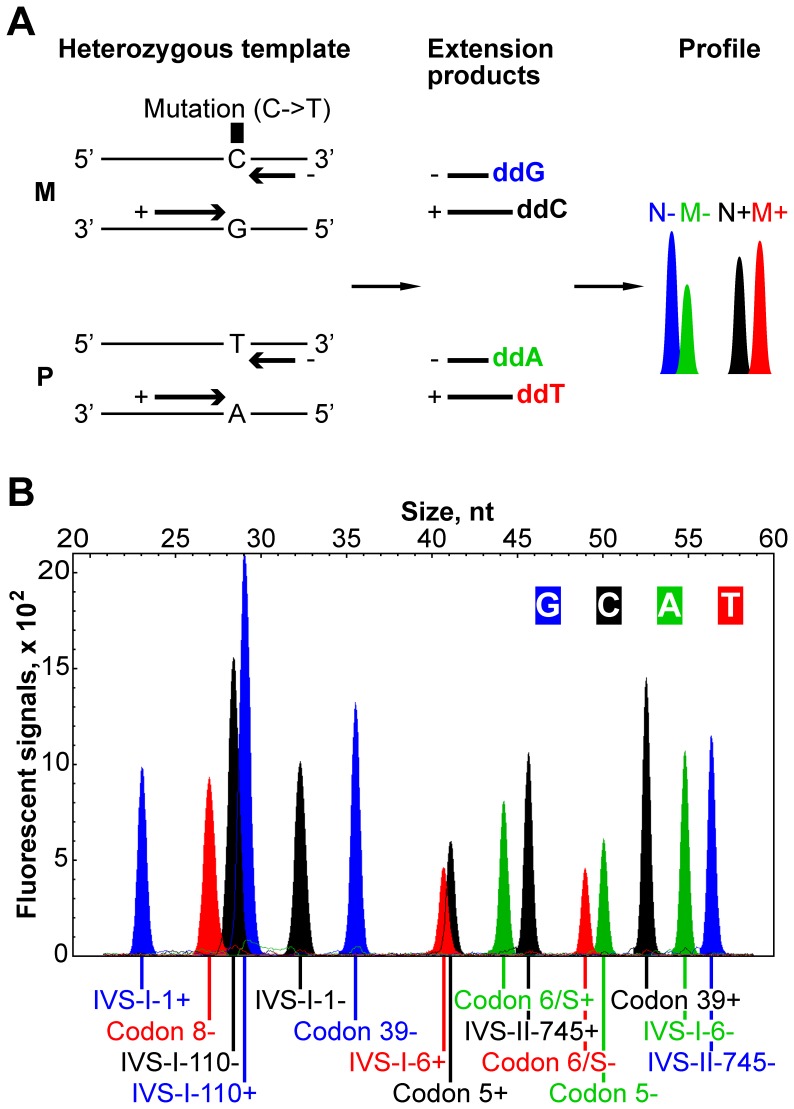
Developing the single-nucleotide primer extension assay. (A) Principle of the single-nucleotide primer extension method illustrated through analysis of a sample carrying a point mutation of interest. Four template DNA strands from the maternal (M) and paternal (P) chromosomes are shown (variable nucleotide lettered). The template is interrogated by two extension primers (thick arrows) giving rise to normal and mutant extension products and peaks. ‘+’ and ‘−’ indicate strand specificity of the primers; the fluorescently labeled nucleotides incorporated into extension products are bold and colored as they appear on the electropherogram. N+, normal peak generated from ‘+’ primer; M+, mutant peak generated from ‘+’ primer; N−, normal peak generated from ‘−’ primer; M−, mutant peak generated from ‘−’ primer. (B) Normal DNA electropherogram profile obtained with the optimized primer set: primer extension product peaks are labeled with the corresponding primer names as in Table 2.

In single-nucleotide extension genotyping, the 3′ end of each primer should be placed immediately adjacent to a variant nucleotide of interest so that normal and mutant genotypes are differentiated by the label of the added terminator. Multiplexing is achieved by mixing primers of different lengths. We reasoned that we would accomplish superior accuracy through interrogating every mutation twice by including two oligonucleotides per mutation, one for each strand (Figure 1A). Our optimized primer set is presented in Table 2. All mutations except Codon 8 (-AA) are cross-examined by a total of 15 primers. The relative sizes of the multiplexed primers determines the order of the extension products on the electropherogram. Although mutation examination by primer extension permits no flexibility with respect to the location of the primers, primer length can be varied to adjust melting temperatures and the potential for formation of hairpins and dimers. Stable oligonucleotide secondary structures and primer dimers can affect signals. Moreover, structures involving primer 3′ ends could result in the formation of template-independent extension products. To minimize these effects, primer self-complementarity and dimerization potential were taken into account during the design process (see Materials and Methods; change in free energy dG values for the final primer set are listed in Table S1). This way the characteristics of the sequences surrounding the mutations impose limitations on primer size and product order (Table 2).

**Table 2:** Primer extension set.

Primer/product number^a^	Primer/product name^b^	Mutation interrogated	Primer sequence	Used at,µM^c^	Product size, nt^d^	N/M genotype^e^
1	IVS-I-1+	IVS-I-1 (G->A)	GGTGAGGCCCTGGGCAG	1.6	18	G/A
2	Codon 8−	Codon 8 (-AA)	ACAGGGCAGTAACGGCAGAC	2.4	21	T/C
3	IVS-I-110−	IVS-I-110 (G->A)	CAGCCTAAGGGTGGGAAAATAGAC	0.3	25	C/T
4	IVS-I-110+	IVS-I-110 (G->A)	ATAGGCACTGACTCTCTCTGCCTATT	0.1	27	G/A
5	IVS-I-1−	IVS-I-1 (G->A)	CTTAAACCTGTCTTGTAACCTTGATACCAA	0.4	31	C/T
6	Codon 39−	Codon 39 (C->T)	(C)_9_ ATCCCCAAAGGACTCAAAGAACCTCT	0.06	36	G/A
7	IVS-I-6+	IVS-I-6 (T->C)	(C)_18_ AGGCCCTGGGCAGGTTGG	2.4	37	T/C
8	Codon 5+	Codon 5 (-CT)	(C)_15_ CAGACACCATGGTGCATCTGACTC	1.2	40	C/G
9	Codon 6/S+	Codon 6 (-A)	(C)_17_ ACACCATGGTGCATCTGACTCCTG	0.8	42	A/G
10	IVS-II-745+	IVS-II-745 (C->G)	(C)_16_ CATATTGCTAATAGCAGCTACAATCCAG	0.8	45	C/G
11	Codon 6/S−	Codon 6 (-A)	(C)_27_ GCAGTAACGGCAGACTTCTCC	0.8	49	T/C
12	Codon 5−	Codon 5 (-CT)	(C)_28_ CAGTAACGGCAGACTTCTCCTC	0.8	51	A/G
13	Codon 39+	Codon 39 (C->T)	(C)_27_ GCTGCTGGTGGTCTACCCTTGGACC	0.4	53	C/T
14	IVS-I-6−	IVS-I-6 (T->C)	(C)_24_ GTCTCCTTAAACCTGTCTTGTAACCTTGAT	0.8	55	A/G
15	IVS-II-745−	IVS-II-745 (C->G)	(C)_26_ TATCCCAACCATAAAATAAAAGCAGAATGGTA	0.1	59	G/C

a Primers are numbered according to the order of the corresponding normal peaks on the electropherogram (Figure 1B).

b Name ending on “+” indicates that primer is identical to the coding strand of the gene (for reference, see *HBB* sequence in NCBI entry NG_000007.3); name ending on “−” signifies identity to the opposite, template strand.

c Final concentration in primer extension reaction.

d Length of extension product in nucleotides (nt) = primer length in nt +1 nt.

e Added terminator for normal (N) and mutant (M) template.

**Table 3:** Reference chromosomes used for validation of the single-nucleotide primer extension assay.

Mutation group	Genotype/mutation name^a^	Genotype calls/total chromosomes tested		Sensitivity, %	Specificity, %
		Presence of mutation	Absence of mutation		
Included in assay	Codon 5 (-CT); CCT(Pro)->C-	5/5	0/5	100	N/A
	Codon 6 (-A); GAG(Glu)->G-G	3/3	0/3	100	N/A
	beta 6(A3) Glu>Val	1/1	0/1	100	N/A
	Codon 8 (-AA); AAG(Lys)->-G	5/5	0/5	100	N/A
	IVS-I-1 (G->A)	11/11	0/11	100	N/A
	IVS-I-6 (T->C)	20/20	0/20	100	N/A
	IVS-I-110 (G->A)	22/22	0/22	100	N/A
	Codon 39 (C->T)	12/12	0/12	100	N/A
	IVS-II-745 (C->G)	5/5	0/5	100	N/A
Not included in assay	−101 (C>T)	0/1	1/1	N/A	100
	−30 (T->A)	0/5	5/5	N/A	100
	Codons 82/83 (-G); AAG·GGC(Lys·Gly)->AAG·-GC	0/1	1/1	N/A	100
	IVS-II-1 (G->A)	0/3	3/3	N/A	100
	IVS-II-848 (C->A)	0/2	2/2	N/A	100
	5′UTR; +22 (G->A)	0/1	1/1	N/A	100
	Poly A (A->G); AAT*A*AA->AAT*G*AA	0/1	1/1	N/A	100
N/A	Normal	0/30	30/30	N/A	100

a Huisman et al. [3].

Our multiplex assay relies on the simultaneous extension of several primers, subsets of which overlap and thus compete with each other. To examine the feasibility of the design, we first tested the primer set on DNA from normal individuals. The electropherograms were highly reproducible showing 15 extension product peaks corresponding to the normal *HBB* sequence and no unexpected peaks (Figure 1B). This indicates that all primers, including competing ones, produce detectable signals implying that the 15-plex primer set can be used for genotyping. Combined with the data on primer secondary structure and dimerization (Table S1), our results provide a comprehensive source of reference for the design of single-nucleotide extension primer mixes.

**Figure 2:**
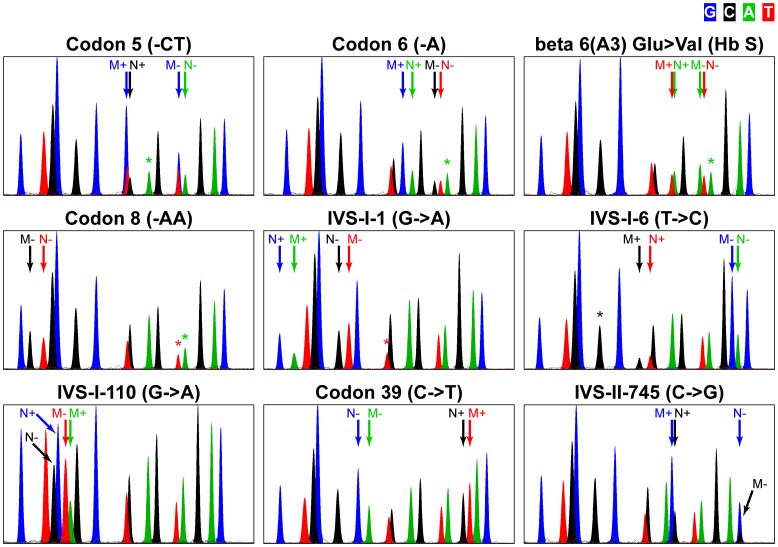
Single-nucleotide primer extension assay performance. Analysis of DNA from heterozygous carriers of the assayed mutations, as indicated above the electropherograms. For each mutation, color-coded arrows denote normal and mutant genotype peaks. ‘+’ and ‘−’ indicate strand specificity of the primers; N+, normal peak generated from ‘+’ primer; M+, mutant peak generated from ‘+’ primer; N−, normal peak generated from ‘−’ primer; M−, mutant peak generated from ‘−’ primer. Asterisks mark peaks that are lower than normal due to interference from genetic variations within the primer-hybridizing template sequence.

We went on to test nine heterozygous samples, each carrying one of the mutations of interest (Figure 2). A specimen heterozygous for one of the interrogated mutations is expected to display two extra peaks (one for Codon 8 (-AA)) in addition to the 15 normal extension products. In most cases, the product from the mutant allele would migrate differently from the normal one, largely due to mass differences between dye-coupled nucleotides. Relative peak height can also vary significantly with the added nucleotide [32]. It is therefore important to confirm that all products, including these generated from mutant alleles, are detected and resolved by capillary electrophoresis. We observed that each mutation is manifested by well defined mutation-specific peaks in the electropherogram (Figure 2; Table S1). Normal genotype peaks are present but reduced in height, as expected for half the normal sequence dosage. These data show that the primer extension assay successfully detects the eight thalassemia mutations and the HbS hemoglobin variant.

We next sought to assess the accuracy of the method by testing pre-genotyped samples, examining the proportion of correctly identified mutations (true positives) as well as the proportion of normal genotype calls obtained with non-carrier specimens (true negatives). We assayed a set of 128 reference chromosomes from normal individuals, mutation carriers and thalassemia major patients. Our results showed 100% agreement with the independently determined genotypes demonstrating that the new assay is highly accurate (Table 3). Taken together, our analyses show that the multiplex assay is suitable for the detection of the nine Mediterranean mutations for diagnostic purposes.

### Sequence Variations do not Compromise the Results of the Multiplex Assay

Polymorphisms and mutations within primer-annealing regions of the template could decrease primer extension efficiency. We wanted to evaluate the effects of variations located near the 3′ ends of extension primers. We noted that the presence of the IVS-I-6 (T->C) mutation creates a mismatch with the fifth nucleotide from the 3′ end of primer IVS-I-1-. Conversely, the IVS-I-1 (G->A) mutation would affect primer IVS-I-6+. Indeed, we observed that the relative height of the IVS-I-1- peak is reproducibly decreased when the IVS-I-6 mutation is present and the peak produced from primer IVS-I-6+ is substantially lower than normal in IVS-I-1 (G->A) samples (Figure 2). These negative effects can be quantified by analyzing IVS-I-1/IVS-I-6 compound heterozygotes whereby the normal IVS-I-1- signal is produced exclusively from the IVS-I-6 (T->C) chromosome and the normal IVS-I-6+ signal is generated from the IVS-I-1 (G->A) chromosome. Our results reveal a several-fold reduction of the IVS-I-1- signal in IVS-I-1/IVS-I-6 compared to normal samples (Figure S2). The negative effect of IVS-I-1 (G->A) on primer IVS-I-6+ is even stronger effectively eliminating the IVS-I-6+ signal (Figure S2). Likewise, each of the three assayed microdeletions perturbs the extension of certain primers in a predictable manner: Codon 5 (-CT) strongly inhibits the signal of primer Codon 6/S+, Codon 6 (-A) interferes with primer Codon 5-, while Codon 8 (-AA) affects both primers Codon 5- and Codon 6/S- (Figure S2, Figure 2). We also tested two samples homozygous for a commonly occurring silent codon 2 polymorphism (Codon 2 (T->C); CAT(His)->CAC(His), HGVS name NG_000007.3:g.70603T>C, Reference SNP ID rs713040) and found that it has no noticeable effect on overlapping primers Codon 5+ and Codon 6/S+ (Figure S2). These data indicate that imperfections in base pairing caused by template sequence variations can have variable effects on assay performance. Notably, certain variations can dramatically affect primer extension signals, especially when located close to the 3′ end of a primer. Nevertheless, these effects do not lead to any ambiguity in genotype calling and thus do not interfere with the analysis of assay results.

### Correct Interpretation of Assay Results Requires that the Presence of Deletions be Determined

Samples heterozygous for *HBB* locus deletions can be genotyped incorrectly by PCR-based assays that fail to amplify the allele carrying the deletion. For example, the sequence hybridizing to the forward primer amplifying the template for the primer extension reaction is located within the common Lepore Boston-Washington deletion [39], [40]. Thus, in Lepore heterozygotes, only the non-Lepore allele would be amplified. To confirm this we tested ten samples carrying the Lepore deletion. As expected, the primer extension pattern of simple Lepore heterozygotes erroneously points to a normal genotype (not shown). Likewise, the profiles of compound heterozygotes carrying both the Lepore deletion and one of the thalassemia mutations included in the primer extension test are identical to these of homozygotes for the thalassemia mutation (Figure S2). These results illustrate the fact that for accurate genotype calling, deletions should be excluded or confirmed independently.

## Discussion

### The Multiplex Assay Provides Substantial Advantages Over Alternative Methods

We aimed to set up a diagnostic protocol that would provide quick and definitive diagnosis for the majority of cases tested in our laboratory. We first evaluated available methodologies taking into account reported accuracy, hands-on time and feasibility. We considered several automatable technologies. Array techniques would have been an attractive option except for the need to produce custom arrays, which requires specialized equipment. Melting curve assays substantially reduce analysis time by eliminating post-PCR steps, however, they also rely on the availability of a high-resolution melting instrument and can produce ambivalent results that require additional testing [24]. In comparison, automated sequencing and primer extension reach higher confidence in mutation calling [41]. Sequencing enables a comprehensive investigation of *HBB* mutations, however, it has its shortcomings. Several reactions have to be run in order to sequence the whole gene. Focusing on key regions can reduce labor and cost but nonetheless, examining the nine mutations requires a minimum of two sequencing reactions per patient, ideally four to sequence both strands and minimize the risk of missing a point mutation in the heterozygous state [11]. The number of reactions can be reduced if multiplex minisequencing is used. In our laboratory, the technique has proved to be highly reliable in several applications [42]–[44]. With that in mind, single-nucleotide extension was deemed to be best suited for our purposes. Well designed primer extension assays provide the desired combination of high accuracy, semi-automation and wide availability of the necessary instrumentation [12], [32]. Mass spectrometry could be used for analysis of the extension products as an alternative to capillary electrophoresis [33], [35] further adding to flexibility. Kobayashi and coauthors [31] and Galbiati et al. [34] have previously described single-nucleotide primer extension assays for the detection of groups of mutations very similar to our mutation set. However, both assays require several extension reactions to cover all mutations. In addition, Galbiati et al. report a relatively low confidence level for assigning genotypes. In contrast, our assay determines all mutations in one reaction and more importantly, utilizes both strands for mutation interrogation reaching very high levels of accuracy, equivalent to the sequencing of both genomic strands. Thus, in comparison with previously published assays for the detection of Mediterranean mutations, our method presents substantial improvement of throughput and precision. The test is highly sensitive and specific, rapid and easy to automate offering a high-quality alternative to other methods.

### Potential Caveats are Carefully Characterized

Any primer-based test can be affected by the presence of sequence variations in primer-annealing sequences. Such effects need to be assessed when evaluating assay reliability. Firstly, in our assay, the PCR primers used for template amplification prior to primer extension are located in regions of low variability to ensure that they amplify various alleles with equal efficiency (see Materials and Methods). Secondly, we have shown that simple sequence variations located close to the mutations of interest can cause deviations in extension peak profiles, such as lower or missing signals. This applies primarily to normal genotype peaks since the normal sequence for a given nucleotide occurs in a variety of different genetic backgrounds. In contrast, there is much less variability within individual minor alleles and thus the ability of the primer set to reproducibly detect the mutant genotype in a number of samples is a good indication that the allele of interest is dependably identified by the assay. Furthermore, in our assay the probability of missing a mutation is virtually eliminated by the introduction of two primers per mutation. Finally, we have confirmed that a common deletion spanning primer sequences impacts the assay in a predictable manner. Other deletions known to occur in the Mediterranean population [3], [39], if undetected, would interfere with the interpretation of assay results indicating that investigations for the presence of deletions should be conducted whenever appropriate [11].

### The New Diagnostic Protocol is Widely Applicable

Our diagnostic procedure targets mutations common throughout the Mediterranean region. According to the available mutation frequency data, the assayed sequence variations together account for most cases of β-hemoglobinopathy in Macedonia (89%), Albania (81%), Bulgaria (82%), Romania (94%), Greece (92%), Cyprus (99%), Spain (81%), France (87%), Italy (86%; 97% in Sicily) as well as substantial numbers of cases in Serbia and Montenegro, Tunisia, Egypt, Turkey and other countries [45]. These data show that the assay can be used as an effective screening tool in routine hemoglobinopathy diagnostics in many countries. In the minority of cases when hematological tests indicate β-hemoglobinopathy and yet the specimen remains undiagnosed by our molecular screen, the sample needs to be further analyzed for less common mutations. Simple modifications to the primer extension set can adapt the assay to particular target populations and minimize these additional analyses. Taken together, our data indicate that the new primer extension assay can be applied across wide geographic areas meeting the highest diagnostic standards.

## Materials and Methods

### Biological Material

We used genomic DNA samples from patients previously genotyped for hemoglobinopathy mutations as described [17], [36], [46]. Several of the reference samples were genotyped by sequencing. DNA was isolated from peripheral blood following standard phenol-chloroform extraction procedures and stored at 4°C. Participants have given written informed consent in accordance with the Declaration of Helsinki. The study has been approved by the Ethics Committee of the Macedonian Academy of Sciences and Arts.

### Primer Design and Optimization

All primer sequences are listed in Table 2 and Table S2. We used the information deposited to the dbSNP database [47] to verify that the primers amplifying the *HBB* fragment encompassing all mutations included in the assay hybridize to invariable sequences.

For the design of the primer extension mix, oligonucleotide melting temperatures were calculated using the RaW-probe program available at www.mlpa.com/support (Table S1). Secondary structures and potential primer dimers were predicted using the UNAFold/mfold server at http://mfold.rna.albany.edu/, applications Homodimer simulations and Hybridization of two different strands [48]–[50]. To achieve electrophoretic resolution of the multiplexed extension products, oligos expected to carry the same label were set to differ by a minimum of 3 nucleotides. Oligo(dC) 5′-non-homologous tails were used to adjust the size of primers longer than 30 nucleotides, keeping the melting temperatures of the sequences complementary to the template within a range of several degrees (Table S1).

All oligonucleotides were synthesized and purified by standard desalting at IDT. The observed length of a labeled extension product can deviate from its actual size since electrophoretic mobility is influenced by both sequence and label. For this reason the identity of the peaks was confirmed experimentally by exclusion of individual primers from the mix. Initially, the relatively large deviation of the mobility of one product (IVS-I-6+) resulted in poor resolution of two fragments carrying identical labels. This was corrected through redesigning the length of several primers and re-running the test to ensure good distribution of the products. Finally, primer concentrations were empirically adjusted to balance peak heights.

### Single-nucleotide Primer Extension

A 1856 bp *HBB* fragment was amplified by PCR (for primer sequences see Table S2) in a mix containing 50 mM Tris-HCl, pH 9.2, 16 mM (NH_4_)_2_SO_4_, 0.1% Tween 20, 2 mM MgCl_2_, 200 µM each dNTP, 200 nM each forward and reverse primer, and 1.2 U Tth polymerase [51] in a final volume of 25 or 50 µl. Cycling conditions were: initial ‘hot-start’ denaturation at 95°C for 10 min followed by 35 cycles of 95°C for 30 s, 58°C for 1 min, 68°C for 1 min 45 s, and a final extension at 68°C for 7 min. A 1.4 µl aliquot of the completed reaction typically containing 5–20 ng specific product was then treated with 0.6 µl Exonuclease I and Shrimp Alkaline Phosphatase mix (ExoSAP-IT, USB) for 15 min at 37°C to eliminate unincorporated nucleotide triphosphates and excess PCR primers. The enzymes were heat-inactivated at 86°C for 20 minutes and the purified PCR product was directly used as template in a primer extension reaction containing the mutation-specific primer cocktail. For the extension reaction, we used the ABI PRISM SNaPshot Multiplex Kit (Life Technologies) following manufacturer’s instructions except that we reduced the reaction volume and diluted the mix supplying the DNA polymerase and fluorescently labeled terminators. The SNaPshot reaction contained 2 µl ExoSAP-treated PCR product, 1 µl 5× primer cocktail (for primer concentrations see Table S2) and 1 µl SNaPshot Multiplex Ready Reaction Mix in a final volume of 5 µl. Primer extension was performed on a thermal cycler for 25 cycles of 96°C for 10 s, 50°C for 5 s, 60°C for 30 s. The extension products were treated with 1 unit Shrimp Alkaline Phosphatase (SAP, USB) at 37°C for 1 hour followed by enzyme inactivation at 65°C for 15 minutes. A 1 µl aliquot of the SAP-inactivated single-nucleotide extension reaction was added to 12 µl HiDi Formamide (Life Technologies) supplied with 0.25 µl GeneScan 120 LIZ Size Standard (Life Technologies). The mixture was denatured at 95°C for 5 minutes, transferred to ice for 2 minutes and loaded onto an ABI PRISM 3010 Genetic Analyzer (Life Technologies). Capillary electrophoresis was performed following manufacturer’s instructions. Extension products were visualized and called automatically using GeneScan 4.0 (Life Technologies).

## Supporting Information

Figure S1 **Point mutations and microdeletions detected by the single-nucleotide primer extension assay.** A map of the human *HBB* gene showing the positions of the beta-thalassemia mutations. Top gene map features: thick rectangles, coding sequences; thin rectangles, untranslated exon sequences; lines, intronic sequences; arrowheads indicate the direction of transcription. A region spanning parts of the first exon and first intron is blown up below the main map: codons are represented by the respective amino acids in single-letter code. Mutations: the positions are indicated by vertical lines (top map) or rectangles (zoomed region) above the gene line labeled with the mutation names.
(TIF)

Figure S2 **Analysis of several reference DNA samples by the single-nucleotide primer extension assay.** Sample genotypes are indicated above the electropherograms. Color-coded labels of normal genotype peaks (top graphs) correspond to primer names (see Table 2). Color-coded arrows denote normal and mutant genotype peaks for the detected mutations; empty arrowheads denote the absence of normal peaks in samples from homozygous patients and Lepore compound heterozygotes. N+, normal peak generated from ‘+’ primer; M+, mutant peak generated from ‘+’ primer; N-, normal peak generated from ‘−’ primer; M-, mutant peak generated from ‘−’ primer. Peaks lower than normal due to interference from genetic variations within the primer-hybridizing template sequence are indicated by a single asterisk, while two asterisks denote undetectable, i.e. significantly affected signals.
(TIF)

Table S1 Single-nucleotide primer extension assay: characterization of primers and products.
(PDF)

Table S2 List of reagents and solutions.
(PDF)
