# Supplementary figures and images for: Efficient Detection of Mediterranean β-Thalassemia Mutations by Multiplex Single-Nucleotide Primer Extension
Source: PLoS One. 2012 Oct 26;7(10):e48167. doi: 10.1371/journal.pone.0048167 (PMC3482202; doi:10.1371/journal.pone.0048167)

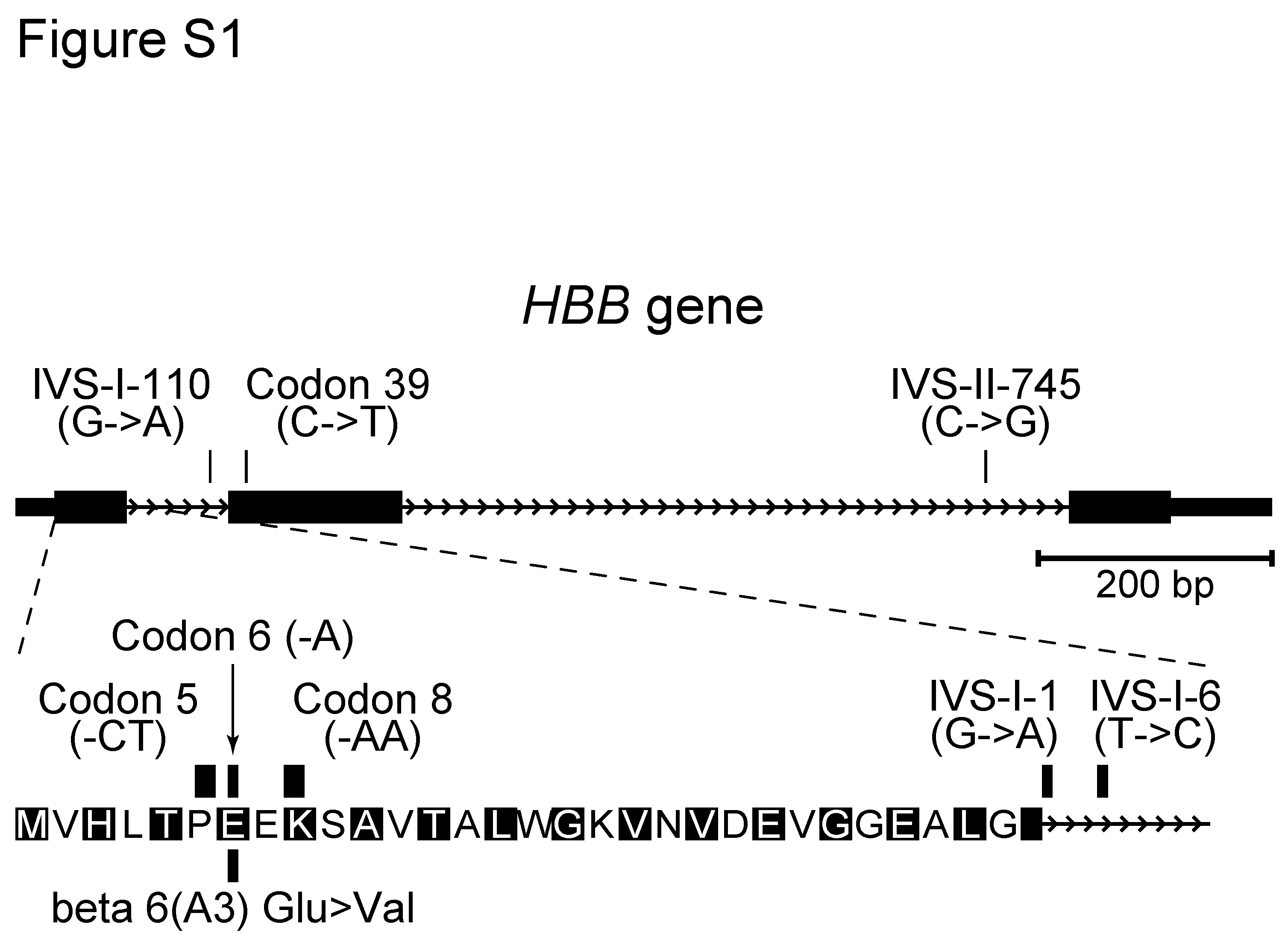

Supplement: Figure S1 — Point mutations and microdeletions detected by the single-nucleotide primer extension assay. A map of the human HBB gene showing the positions of the beta-thalassemia mutations. Top gene map features: thick rectangles, coding sequences; thin rectangles, untranslated exon sequences; lines, intronic sequences; arrowheads indicate the direction of transcription. A region spanning parts of the first exon and first intron is blown up below the main map: codons are represented by the respective amino acids in single-letter code. Mutations: the positions are indicated by vertical lines (top map) or rectangles (zoomed region) above the gene line labeled with the mutation names. (TIF) [file pone.0048167.s001.tif]

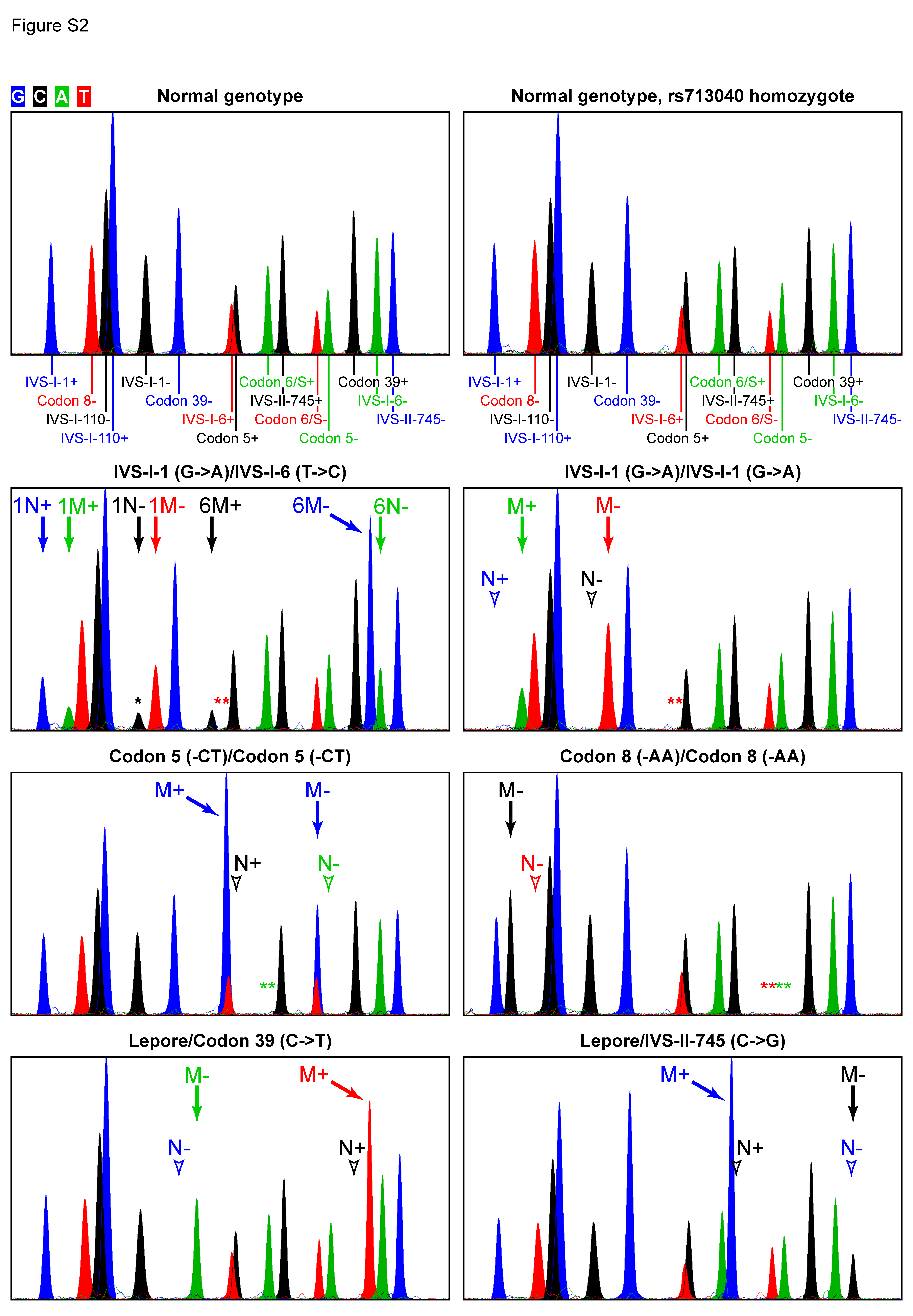

Supplement: Figure S2 — Analysis of several reference DNA samples by the single-nucleotide primer extension assay. Sample genotypes are indicated above the electropherograms. Color-coded labels of normal genotype peaks (top graphs) correspond to primer names (see Table 2). Color-coded arrows denote normal and mutant genotype peaks for the detected mutations; empty arrowheads denote the absence of normal peaks in samples from homozygous patients and Lepore compound heterozygotes. N+, normal peak generated from ‘+’ primer; M+, mutant peak generated from ‘+’ primer; N-, normal peak generated from ‘−’ primer; M-, mutant peak generated from ‘−’ primer. Peaks lower than normal due to interference from genetic variations within the primer-hybridizing template sequence are indicated by a single asterisk, while two asterisks denote undetectable, i.e. significantly affected signals. (TIF) [file pone.0048167.s002.tif]
