# Supplementary material for: Efficient Detection of Mediterranean β-Thalassemia Mutations by Multiplex Single-Nucleotide Primer Extension
Source: PLoS One. 2012 Oct 26;7(10):e48167. doi: 10.1371/journal.pone.0048167 (PMC3482202; doi:10.1371/journal.pone.0048167)
Supplement: Table S1 — Single-nucleotide primer extension assay: characterization of primers and products. (PDF) [file pone.0048167.s003.pdf]

Table S1. Single-nucleotide primer extension assay: characterization of primers and products.

Primer/product number	Primer/product name	Primer size, nt	Complementary sequence size, nt[a]	Tm, °C[b]	Secondary structure, dG, kcal/mol[c]	Homodimer, dG, kcal/mol[d]	Heterodimer with primer(s), dG, kcal/mol[e]	Normal peak[f]	Mutant peak[g]
1	IVS-I-1+	17	17	63.6	-0.34	-7.8	IVS-I-6+, -8.2	G 23.3	A 26.0
2	Codon 8-	20	20	59.4	1.08	-1.8	IVS-I-110+, -6.0<br>IVS-I-6+, -6.0<br>IVS-I-1+, -6.0	T 27.3	C 23.8
3	IVS-I-110-	24	24	61.1	0.56	-4.0	IVS-II-745-,-5.2	C 28.6	T 30.0
4	IVS-I-110+	26	26	59.0	-1.46	-3.4	Codon 6/S-, -4.4<br>Codon 5-, -4.4	G 29.3	A 31.4
5	IVS-I-1-	30	30	60.8	1.55 (E)	-0.1 (E)	IVS-I-6+, -4.2	C 32.5	T 34.2
6	Codon 39-	35	26	62.4	0.13	-1.9	IVS-I-110-, -4.2	G 35.7	A 37.7
7	IVS-I-6+	36	18	63.9	-1.53	-8.6	IVS-I-110-, -4.5	T 41.1	C 39.3
8	Codon 5+	39	24	63.8	0.18	-8.1	Codon 6/S+, -8.6	C 41.3	G 40.6
9	Codon 6/S+	41	24	63.6	0.18	-8.1	IVS-I-110-, -4.6	A 44.3	G 42.6
10	IVS-II-745+	44	28	59.0	-0.10	-3.4	Codon 39+, -5.2	C 45.8	G 45.2
11	Codon 6/S-	48	21	59.8	-0.19	-1.7	Codon 39+, -3.7	T 49.1	C 48.1
12	Codon 5-	50	22	59.2	1.34	-1.0	Codon 39+, -3.7	A 50.0	G 49.2
13	Codon 39+	52	25	69.9	0.18 (E)	-1.6 (E)	IVS-I-110-, -4.6	C 52.7	T 53.7
14	IVS-I-6-	54	30	61.8	2.01	0.1	IVS-I-6+, -4.2	A 54.9	G 54.1
15	IVS-II-745-	58	32	60.8	0.36	-3.0	IVS-I-6+, -4.4 (E)<br>Codon 39+, -4.4	G 56.5	C 56.5

[a] Size of sequence complementary to template DNA (= primer size – size of poly(C) tail)

[b] Melting temperature for complementary sequence calculated using RaW; Tm conditions: Salt Concentration 0.05 M; Oligo Concentration 0.1 μM; Calculation Method Go-Oli-Go

[c] Secondary structure predictions made using the mfold server with Folding temperature set at 50°C and Ionic conditions: [Na^+] at 0.05 M, [Mg^{++}] at 0.0025 M; dG is the free energy released (negative values) or absorbed (positive values) in the process of forming the most favored secondary structure; (E) indicates that the structure is extendable, i.e. the '3-end nucleotide is included in the hairpin stem

[d] Dimer predictions made using the UNAFold server with temperature set at 50°C, [Na^+] = 0.05 M, [Mg^{++}] = 0.0025 M, Strand concentration at 2.4 μM; (E) indicates extendability

[e] Dimer predictions as detailed in footnote d; the dimer with the lowest dG is listed; second lowest is listed if the lowest combination is already presented; (E) indicates extendability

[f] Electropherogram details of the normal genotype signal: added 3'-end nucleotide is color-coded to match peak color; average observed peak size is given in nt

[g] Electropherogram details of the mutant genotype signal, as in footnote f
