# Supplementary material for: Efficient Detection of Mediterranean β-Thalassemia Mutations by Multiplex Single-Nucleotide Primer Extension
Source: PLoS One. 2012 Oct 26;7(10):e48167. doi: 10.1371/journal.pone.0048167 (PMC3482202; doi:10.1371/journal.pone.0048167)
Supplement: Table S2 — List of reagents and solutions. (PDF) [file pone.0048167.s004.pdf]

Table S2. List of reagents and solutions.

Reagent/solution	Used for	Supplier, details, comments
Primer 5'-GCCAAGGACAGGTACGGCTGTCATC-3'	PCR amplification of 1856 bp *HBB* fragment – forward primer	Any supplier of synthetic DNA oligonucleotides, purification by standard desalting
Primer 5'-GTATTTTCCCAAGGTTTGAACTAGCTC-3'	PCR amplification of 1856 bp *HBB* fragment – reverse primer	Any supplier of synthetic DNA oligonucleotides, purification by standard desalting
Thermostable DNA polymerase with reaction buffer and $MgCl_2$ stock solution	PCR amplification of 1856 bp *HBB* fragment	Any supplier of molecular biology products
dATP, dCTP, dGTP, dTTP	PCR amplification of 1856 bp *HBB* fragment	Any brand, PCR grade
Agarose	Quality control of PCR product by agarose electrophoresis (optional)	Any brand, molecular biology grade
TBE buffer (10 × TBE: 900 mM Tris, 900 mM boric acid, 20 mM EDTA)	Quality control of PCR product by agarose electrophoresis (optional)	Prepare stock solution
ExoSAP-IT	PCR product clean-up	GE Healthcare USB, Product Number 78200
Extension primer set: a total of 15 separate oligonucleotides[a]	Primer extension	Any supplier of synthetic DNA oligonucleotides, purification by standard desalting
5 × Primer cocktail: 8 μM IVS-I-1+, 12 μM Codon 8-, 1.5 μM IVS-I-110-, 0.5 μM IVS-I-110+, 2 μM IVS-I-1-, 0.3 μM Codon 39-, 12 μM IVS-I-6+, 6 μM Codon 5+, 4 μM Codon 6/S+, 4 μM IVS-II-745+, 4 μM Codon 6/S-, 4 μM Codon 5-, 2 μM Codon 39+, 4 μM IVS-I-6-, 0.5 μM IVS-II-745-[b]	Primer extension	Prepare stock solution
ABI PRISM SNaPshot Multiplex Kit	Primer extension	Life Technologies, Product Number 4323159A
Shrimp Alkaline Phosphatase with reaction buffer	Primer extension	Affymetrix USB, Product Number 70092 or equivalent
HiDi Formamide	Analysis of extension products	Life Technologies, Product Number 4311320
GeneScan 120 LIZ Size Standard	Analysis of extension products	Life Technologies, Product Number 4322362
Consumables for fragment analysis by capillary electrophoresis	Analysis of extension products	Follow the recommendations of the instrument manufacturer

[a] Primer sequences listed in Table 2

[b] Primer names as in Table 2
